# F18. IS SCHIZOPHRENIA A MULTI-SYSTEM DISORDER? CONSIDERING NEUROLOGICAL, IMMUNE, CARDIOMETABOLIC, AND ENDOCRINE ALTERATIONS IN FIRST EPISODE PSYCHOSIS

**DOI:** 10.1093/schbul/sby017.549

**Published:** 2018-04-01

**Authors:** Toby Pillinger, Enrico D’Ambrosio, Rob McCutcheon, Oliver Howes

**Affiliations:** 1 Institute of Psychiatry, King’s College; 2 King’s College London, Institute of Psychiatry; Medical Research Council Clinical Sciences Centre, Hammersmith Hospital, Imperial College

## Abstract

**Background:**

People with schizophrenia and related psychotic disorders show abnormalities in several organ systems in addition to the central nervous system (CNS). It is not yet known how the magnitude of disturbance in non-CNS systems compares with the magnitude of CNS disturbance in FEP. Here, we statistically compare effect sizes (ES) for non-CNS and CNS dysfunction in FEP, and consider whether schizophrenia is a multi-system disorder.

**Methods:**

Pubmed was systematically searched from 1990 to May 2017 for meta-analyses examining non-CNS dysfunction in FEP, focusing on immune, cardiometabolic, and hypothalamic-pituitary-adrenal (HPA) systems. A parallel search was performed for meta-analyses examining representative CNS dysfunction in FEP, specifically neurophysiological, neurochemical and brain structural alterations. To statistically compare the magnitude of effect sizes (ES) between different CNS and non-CNS systems in FEP, data were extracted from case-control studies making up these meta-analyses, and meta-analyses repeated. For non-CNS parameters, random-effects meta-analyses were performed examining immune (cytokines, CRP, and lymphocytes), cardiometabolic (glucose/insulin and lipids), and HPA parameters (cortisol and prolactin). For CNS parameters, meta-analyses were performed examining brain structural, neurophysiological (Auditory P300 amplitude and latency, duration deviant mismatch negativity), and neurochemical parameters (N-acetylaspartic acid levels). Standardized mean differences between patient and control cohort parameters were used as the ES (Hedges adjusted g). As well as individual meta-analyses being run for each parameter as described, 6 separate sub-group meta-analyses were performed examining data for overall immune, cardiometabolic, HPA, brain structural, neurophysiological, and neurochemical systems. Sub-group summary effect size magnitudes were calculated by running a combined analysis of all studies assigned to a sub-group (e.g. to calculate the summary effect size magnitude for immune alterations, a single analysis was performed that combined IL-1b, sIL-2R, IL-6, TNFa, TGFb, CRP, and lymphocyte count data sets). Summary effect sizes for these 6 individual systems were statistically compared in a fixed effects model using a Wald-type test. This was also used to compare overall summary CNS and non-CNS effect sizes. Antipsychotic naïve sensitivity analyses were performed.

**Results:**

Data were extracted for 165 studies comprising a total sample size of 13,440. The summary effect size for immune alterations (g=1.19; CI:0.82–1.56) was significantly greater than brain structural (g=0.40;P<.001) and neurochemical alterations (g=0.43;P<.001), and no different from neurophysiological alterations (g=0.80; P=0.05). The summary effect size for HPA alterations (g=0.68; CI:0.32–1.04) was not significantly different from brain structural (P=.14), neurophysiological (P=.54), and neurochemical (P=.22) alterations. The summary effect size for cardiometabolic alterations (g=0.23; CI:0.15–0.31) was significantly lower than neurochemical (P=.04), neurophysiological (P<.001) and brain structural alterations (P=.001). The overall summary effect sizes for non-CNS (g=0.58; CI:0.44–0.72) and CNS (g=0.50; CI:0.44–0.56) alterations were not significantly different (P=.28).

**Discussion:**

These data indicate that there are robust alterations in non-CNS systems in psychosis, and that these are broadly similar in magnitude to a range of CNS alterations, indicating that psychosis involves multiple organ systems to a comparable degree from onset. We consider three models that could account for these findings and discuss implications for future research and treatment.

